# Composition and dynamics of the respiratory tract microbiome in intubated patients

**DOI:** 10.1186/s40168-016-0151-8

**Published:** 2016-02-11

**Authors:** Brendan J. Kelly, Ize Imai, Kyle Bittinger, Alice Laughlin, Barry D. Fuchs, Frederic D. Bushman, Ronald G. Collman

**Affiliations:** Department of Medicine, Perelman School of Medicine, University of Pennsylvania, Philadelphia, PA 19104 USA; Department of Microbiology, Perelman School of Medicine, University of Pennsylvania, Philadelphia, PA 19104 USA

**Keywords:** Human microbiome, Respiratory failure, Mechanical ventilation, Pneumonia, Ventilator-associated pneumonia, Antibiotics

## Abstract

**Background:**

Lower respiratory tract infection (LRTI) is a major contributor to respiratory failure requiring intubation and mechanical ventilation. LRTI also occurs during mechanical ventilation, increasing the morbidity and mortality of intubated patients. We sought to understand the dynamics of respiratory tract microbiota following intubation and the relationship between microbial community structure and infection.

**Results:**

We enrolled a cohort of 15 subjects with respiratory failure requiring intubation and mechanical ventilation from the medical intensive care unit at an academic medical center. Oropharyngeal (OP) and deep endotracheal (ET) secretions were sampled within 24 h of intubation and every 48–72 h thereafter. Bacterial community profiling was carried out by purifying DNA, PCR amplification of 16S ribosomal RNA (rRNA) gene sequences, deep sequencing, and bioinformatic community analysis. We compared enrolled subjects to a cohort of healthy subjects who had lower respiratory tract sampling by bronchoscopy. In contrast to the diverse upper respiratory tract and lower respiratory tract microbiota found in healthy controls, critically ill subjects had lower initial diversity at both sites. Diversity further diminished over time on the ventilator. In several subjects, the bacterial community was dominated by a single taxon over multiple time points. The clinical diagnosis of LRTI ascertained by chart review correlated with low community diversity and dominance of a single taxon. Dominant taxa matched clinical bacterial cultures where cultures were obtained and positive. In several cases, dominant taxa included bacteria not detected by culture, including *Ureaplasma parvum* and *Enterococcus faecalis*.

**Conclusions:**

Longitudinal analysis of respiratory tract microbiota in critically ill patients provides insight into the pathogenesis and diagnosis of LRTI. 16S rRNA gene sequencing of endotracheal aspirate samples holds promise for expanded pathogen identification.

**Electronic supplementary material:**

The online version of this article (doi:10.1186/s40168-016-0151-8) contains supplementary material, which is available to authorized users.

## Background

Approximately 7.6 % of patients admitted to the hospital with community-acquired pneumonia (CAP) develop respiratory failure requiring mechanical ventilation [1, 2]. Similar proportions of patients who acquire pneumonia during hospitalization (HAP) require mechanical ventilation (5.9 %) [3]. Patients hospitalized with CAP or who develop HAP during hospitalization have a poor prognosis, with a median mortality of 10–12 % [4–6]. Lower respiratory tract infection (LRTI) is also an important complication of mechanical ventilation among patients who develop respiratory failure from other causes. Approximately 15 % of intubated patients receive antibiotics for clinically diagnosed ventilator-associated pneumonia (VAP) or tracheobronchitis [7]. Patients with VAP have an increased length of stay and may have as much as twofold greater mortality than intubated patients without LRTI [8–14].

The dynamics of the full microbial populations in the respiratory tract of intubated patient remain poorly understood. Culture-based analyses of bacterial communities during the course of mechanical ventilation suggest that enteric gram-negative rods and *Pseudomonas* species commonly become dominant over time, but such studies also demonstrate heterogeneity between subjects and timepoints [15–20]. However, routine clinical culture identifies only a subset of the respiratory tract community members. Culture-based analysis has limited sensitivity for fastidious organisms and anaerobes.

16S ribosomal RNA (rRNA) gene sequencing has allowed culture-independent characterization of the bacterial communities in health and disease, termed the microbiome [21–26]. Recently, 16S rRNA gene sequencing has been used for pathogen identification in LRTI [27–29]. However, the respiratory tract microbiome has not been extensively studied in intubated subjects. Here, we performed longitudinal sampling and 16S rRNA gene sequencing of samples from the upper and lower respiratory tract sites from critically ill subjects who were intubated and dependent on mechanical ventilation. Our goals were to (1) define the dynamics of the respiratory tract bacterial microbiome during mechanical ventilation, (2) identify features of bacterial community structure associated with LRTI, (3) assess the correlation between LRTI pathogens identified by clinical culture and 16S rRNA gene sequencing, and (4) detect dominant bacterial species that may not be not recognized by culture.

## Results

### Subject characteristics

**Fig. 1:**
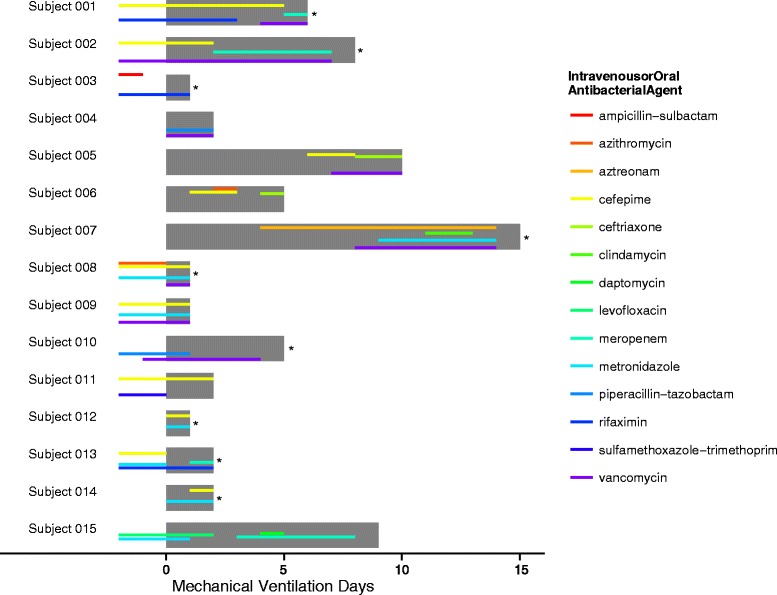
Subject enrollment and antibiotic exposure. Fifteen recently intubated subjects were enrolled in the study. The *horizontal*, *gray bars* depict the duration of each subject’s enrollment: longitudinal data was collected for ten of the 15 enrolled subjects. *Thinner horizontal lines* indicate the intravenous or oral antibiotic exposure of each subject, with line color representing antibiotic identity, from 2 days prior to enrollment through the end of sample collection. The color corresponding to each antibiotic is summarized to the right. *Asterisks* indicate subjects who died during the ICU admission

The 15 enrolled subjects included a heterogeneous set of underlying diseases and acute indications for mechanical ventilation (Table 1). Four subjects had CAP/HAP at enrollment (one of whom was documented to have respiratory syncytial virus (RSV) infection); four were suspected of having aspiration at enrollment (for subjects 4 and 13, aspiration was listed as the most likely diagnosis). Four subjects were given the clinical diagnosis of VAP, and two subjects were suspected of having recurrent aspiration during the course of mechanical ventilation. Intravenous antibacterial agent exposure was extensive and also varied among subjects (Fig. 1). Ten subjects were sampled at multiple timepoints. A total of 42 upper and 42 lower respiratory tract communities were analyzed by 16S rRNA gene PCR and deep sequencing, yielding approximately 5.8 × 10^6^ total reads (median 6.0 × 10^4^ reads per community). Despite the subjects’ clinical heterogeneity, we observed several features that distinguished respiratory tract bacterial community change during mechanical ventilation.

**Table 1:** Subject characteristics and pneumonia diagnosis

Subject	Age	Sex	Chronic disease	Acute diagnosis	Days enrolled	CAP/HAP	VAP	Aspiration at enrollment	Subsequent aspiration
1	61	F	HCV cirrhosis	Sepsis (cx negative)	6	N	Y	Y	Y
2	25	F	AML post allo-BMT	Neutropenic sepsis (cx negative)	8	N	Y	N	N
3	61	M	Cardiomyopathy	PE + PEA arrest	1	N	N	N	–
4	32	F	Epilepsy	Seizure + aspiration	1	N	N	Y	–
5	77	F	ALS	Hypercarbia	10	N	Y	N	N
6	84	F	Metastatic NSCLC	Hypercarbia	5	N	N	Y	Y
7	42	F	Morbid obesity + OHS	Hypercarbia	15	N	Y	N	N
8	65	M	Hypertension	Acute AML	1	N	N	N	–
9	54	F	Asthma	Osteomyelitis + sepsis (cx negative)	1	Y	N	Y	–
10	69	F	ESRD on HD	MRSA sepsis	5	Y	N	N	N
11	60	M	COPD post OLT	Hypoxemia	2	N	N	N	N
12	65	F	AML post allo-BMT + GVHD	Sepsis (cx negative)	1	Y	N	Y	–
13	61	M	EtOH/HCV cirrhosis	Aspiration + sepsis (cx negative)	2	N	N	Y	N
14	49	F	Pancreatic cancer	Obtundation	2	N	N	N	N
15	57	F	Myelodysplastic syndrome	RSV pneumonia	9	Y	N	N	N

Age, sex, underlying disease, and acute indication for mechanical ventilation are described for each enrolled subject. Cases of sepsis without a pathogen identified by blood culture are indicated (cx negative). The clinical diagnosis of LRTI, as ascertained by chart review of the MICU attending physician’s daily progress note, is presented for each subject. LRTI diagnosed within the first 48 h of intubation is categorized as CAP or HAP; LRTI diagnosed after 48 h of intubation is categorized as VAP [42]. Aspiration, likewise ascertained by chart review, is included and categorized as either a suspected contributor to the respiratory failure requiring intubation or as a complication occurring during the course of mechanical ventilation

*HCV* hepatitis C virus, *AML* acute myelocytic leukemia, *BMT* bone marrow transplantation, *PE* pulmonary embolism, *PEA* pulseless electrical activity, *ALS* amyotrophic lateral sclerosis, *NSCLC* non-small cell lung cancer, *OHS* obesity hypoventilation syndrome, *ESRD* end-stage renal disease, *HD* hemodialysis, *COPD* chronic obstructive pulmonary disease, *OLT* orthotopic lung transplantation, *GVHD* graft-versus-host disease, *EtOH* alcohol, *RSV* respiratory syncytial virus

### Intubated subjects have distinct lower respiratory tract bacterial communities compared to healthy controls

**Fig. 2:**
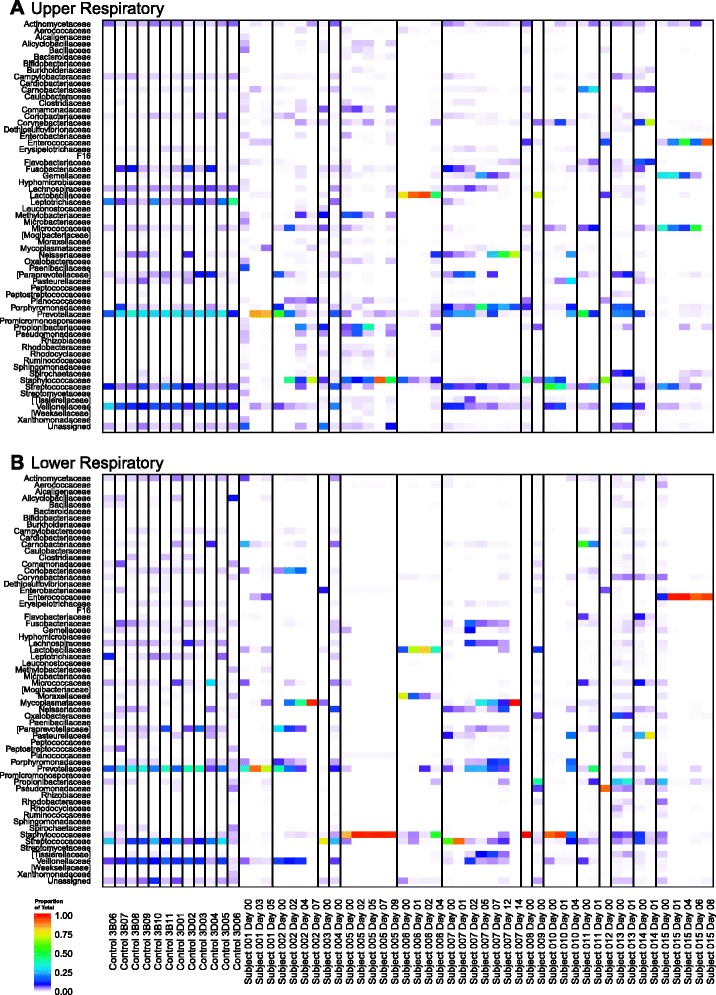
Upper and lower respiratory tract bacterial communities of intubated subjects and healthy controls. Heatmaps for **a** upper and **b** lower respiratory samples are depicted. Each *column* represents a single sample, and each *row* represents family-level taxonomic assignment of the sample’s 16S rRNA gene sequences. *Vertical lines* separate the healthy controls and each intubated subjects. The *color* indicates proportional abundance of the sequences assigned to each bacterial family within the sample

Healthy control subjects were all similar to one another in both upper respiratory tract (URT) and lower respiratory tract (LRT) bacterial communities (Fig. 2a, b). Healthy subjects’ URT and LRT communities were similar within each individual, dominated by the families *Prevotellaceae*, *Streptococcaceae*, and *Veillonellaceae* [30, 31].

In contrast, we observed greater heterogeneity among intubated subjects. Both the upper and lower respiratory tract communities were dominated by specific taxa at most timepoints. Within a given subject and given sampling site, the taxon with highest proportional abundance remained largely consistent across longitudinal samples. Unlike healthy controls, some intubated subjects had different taxa dominating the URT versus LRT communities at a given time.

To evaluate the similarity of LRT bacterial communities within subjects over time, we carried out a statistical comparison of ecological distances, comparing distances across timepoints within subjects to between-subject distances. We first calculated unweighted and weighted Jaccard and UniFrac distances between each pair of LRT samples. We then applied permutational multivariate analysis of variance (PERMANOVA) testing to the pairwise distances to determine the proportion of distance between samples accounted for by subject identity [32]. The coefficient of determination (*R*^2^) and corrected coefficient of determination (*ω*^2^) indicated that among intubated subjects, a large proportion of the distance between samples is accounted for by subject identity—i.e., that samples from the same subject are similar over time—regardless of the distance metric chosen: *R*^2^ 0.506 and *ω*^2^ 0.245 for unweighted (binary) Jaccard, *R*^2^ 0.845 and *ω*^2^ 0.760 for weighted Jaccard, *R*^2^ 0.520 and *ω*^2^ 0.266 for unweighted UniFrac, and *R*^2^ 0.604 and *ω*^2^ 0.394 for weighted UniFrac distances [33]. In all cases, the proportion of distance accounted for by subject identity was highly significant (*p* < 0.001). Thus, we confirmed that longitudinal samples of intubated subjects’ LRT bacterial communities are more similar to each other than to the LRT bacterial communities of other subjects.

### A single OTU dominates the respiratory tract community of many intubated subjects

**Fig. 3:**
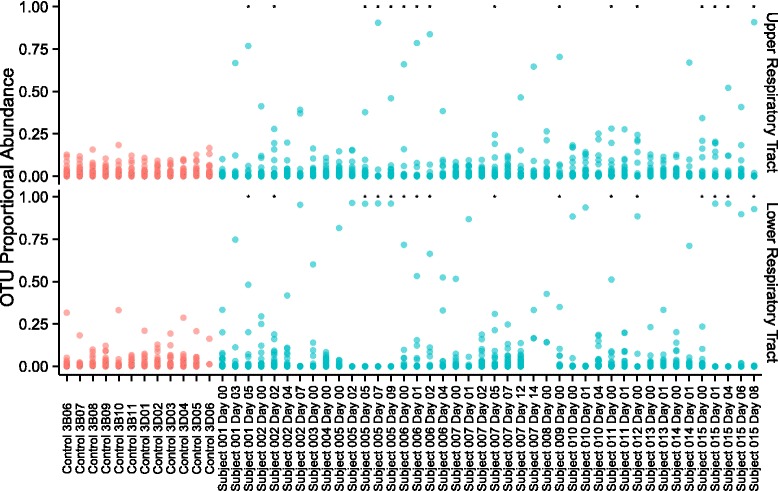
Proportional abundance of bacterial community members in intubated subjects and healthy controls. Each *point* represents an OTU; the samples from which the OTUs were identified are arrayed along the *horizontal axis*, including both upper and lower respiratory tract sites; the proportional abundance of each OTU in the sample from which it was identified is indicated by its position along the *vertical axis*. All OTUs that accounted for >200 reads across all samples are included; OTUs from healthy controls are colored *red*; OTUs from intubated subjects are colored *blue. Asterisks* indicate samples with concurrent documentation of suspected pneumonia by the critical care attending physician

**Fig. 4:**
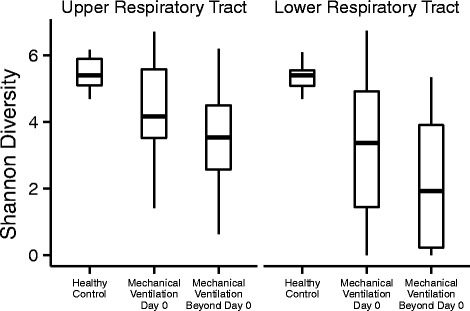
Alpha diversity of intubated subjects over time. Within-sample (alpha) diversity, measured by the Shannon index, is shown for upper (OP) and lower (ET for intubated subjects, BAL for healthy controls) respiratory tract sites. Comparison is made between the diversity of communities in healthy controls, versus the communities of intubated subjects within 24 h of intubation or more than 24 h post intubation

Figure 3 depicts the proportion of the total bacterial community within each respiratory tract sample that is contributed by each taxon. These data suggest that the proportion of reads attributed to the operational taxonomic unit (OTU) with the highest abundance was greater in samples from intubated subjects than healthy controls. To evaluate this relationship while accounting for the longitudinal nature of the data and clustering within subjects, we developed a generalized estimating equation (GEE) model for proportional abundance of the most abundant OTU versus healthy/intubated status. We found that intubated status was associated with a higher maximum OTU proportional abundance in both the upper and lower respiratory tracts (*p* = 1.3e−7 and *p* = 5.6e−11). Consistent with this result, GEE modeling also confirmed that within-sample (alpha) diversity, measured by the Shannon Index, was lower in intubated subjects than healthy controls (*p* = 4.8e−8 and *p* = 2.3e−13 for the URT and LRT, respectively) (Fig. 4).

The within-sample (alpha) diversity was observed to be lower in intubated subjects than healthy controls. In contrast, the between-sample (beta) diversity was greater among intubated subjects than among healthy controls (Additional file 1: Figure S1). Healthy subjects were observed to have similar respiratory tract bacterial community composition, but intubated subjects to have respiratory tract bacterial communities that differ from both healthy subjects and other intubated subjects. We also evaluated the distances between paired oropharyngeal (OP) and endotracheal (ET) bacterial communities in all subjects, but no consistent pattern was evident. The OP-ET distance was not significantly different between subjects with suspected aspiration and those without.

### Upper and lower respiratory tract bacterial community diversity diminishes during the course of intubation

Inspection of heat maps (Fig. 2a, b) suggested loss of diversity with time in some subjects, consistent with dominance of specific bacterial lineages. We investigated the change in diversity statistically using a GEE model relating alpha diversity to days post intubation, and we found that Shannon diversity decreased with time in both the upper and lower respiratory tract. The decrease achieved statistical significance in the upper but not lower respiratory tract (*p* = 0.0015 and *p* = 0.13, respectively, for time as a continuous variable; *p* = 0.015 and *p* = 0.073, respectively, for time categorized as in Fig. 4—i.e., “Day 0” versus “Beyond Day 0”). These observations are consistent with dominance of a single taxon.

### Antibiotic exposure does not fully account for the association between duration of intubation and lower respiratory tract community diversity

Given the potential for antibiotic exposure to confound or modify the relationship between respiratory tract alpha diversity and duration of intubation, we investigated antibiotic effects quantitatively. We incorporated antibiotic exposure (Fig. 1), coded by presence or absence of each antibiotic functional class at each timepoint, into the GEE model described above. No single antibiotic class or combination of antibiotic classes achieved significance by Wald testing. Comparison of GEE models including versus excluding antibiotics by model QIC favored excluding antibiotics [34]. This evidence suggests that the relationship between longer time on a ventilator and lower diversity cannot be attributed solely to greater antibiotic exposure with longer duration of intubation. However, the heterogeneous antibiotic regimens observed in this study (Fig. 1) may contribute to this finding.

### Low lower respiratory tract community diversity is associated with clinical LRTI diagnosis

We observed several subjects with extremely low diversity within 48 h of enrollment (subjects 5, 8, 10, 12, and 15) and sought to evaluate how this pattern related to the diagnosis of LRTI. Chart review was used to capture the clinical diagnosis of LRTI because there is no “gold standard” for LRTI diagnosis that could be applied at each timepoint (the Center for Disease Control and Prevention surveillance criteria for ventilator-associated complication [35] can only be applied after 48 h of stable or improving mechanical ventilation parameters and so do not serve for all samples here). Figure 3 indicates whether LRTI was suspected at each timepoint.

Among intubated subjects, we found that the clinical diagnosis of pneumonia was associated with further reduction in Shannon diversity of the lower respiratory tract, relative even to those subjects with prolonged courses of intubation, though this GEE model did not achieve statistical significance with our small sample size (*p* = 0.08 for LRTI diagnosis documentation analyzed at each time point, as shown in Fig. 3). Simple comparison of respiratory tract Shannon diversity between timepoints with pneumonia and subjects without suspected pneumonia did achieve statistical significance at both LRT and URT (Wilcoxon rank-sum test *p* = 0.0036 and *p* = 0.042, respectively).

### Pathogens identified by clinical culture correlate with assignments from 16S rRNA gene sequence

Bacterial cultures of the lower respiratory tract were obtained during enrollment in 13 subjects and yielded pathogenic bacteria in four subjects, allowing comparison to the results of 16S rRNA gene sequencing. In each of these four cases, 16S rRNA gene sequencing identified the same bacterial species as the most abundant OTU at one or more timepoint (Table 2). In three of the four subjects, positive cultures confirmed the clinical diagnosis of LRTI (Table 1). These included two cases of *Staphylococcus aureus* and one of *Pseudomonas aeruginosa*. In each of these cases (subjects 5, 10, and 12), 16S rRNA gene sequencing identified the same bacterial species as the most abundant OTU at all timepoints. In one of these cases (subject 5), 16S rRNA gene sequencing identified dominance of the pathogenic taxon five days before clinical culture.

**Table 2:** Proportional abundance and taxonomic assignment of dominant OTU at each timepoint

Subject	DPI	Abundance	BLAST assignment	Culture
1	0	0.334	*Prevotella denticola*	Rare yeast
1	3	0.748	*Prevotella denticola*	NA
1	5	0.482	*Prevotella denticola*	*Candida albicans*
2	0	0.296	*Prevotella melaninogenica*	NA
2	2	0.190	*Ureaplasma parvum*	“Few mouth flora”
2	4	0.419	*Ureaplasma parvum*	“Few mouth flora”
2	7	0.954	*Ureaplasma parvum*	NA
3	0	0.602	*Streptococcus salivarius subsp. thermophilus*	NA
4	0	0.084	*Neisseria sicca*	“Few mouth flora”
5	0	0.816	*Staphylococcus aureus subsp. aureus*	NA
5	2	0.965	*Staphylococcus aureus subsp. aureus*	NA
5	5	0.960	*Staphylococcus aureus subsp. aureus*	Many MRSA; few *Klebsiella pneumoniae*
5	7	0.962	*Staphylococcus aureus subsp. aureus*	NA
5	9	0.960	*Staphylococcus aureus subsp. aureus*	NA
6	0	0.717	*Moraxella catarrhalis*	NA
6	1	0.533	*Lactobacillus gasseri*	NA
6	2	0.664	*Lactobacillus gasseri*	Few MSSA; few yeast
6	4	0.525	*Staphylococcus aureus subsp. aureus*	NA
7	0	0.517	*Streptococcus dentisani*	“Rare mouth flora”
7	1	0.869	*Streptococcus dentisani*	NA
7	2	0.189	*Prevotella melaninogenica*	NA
7	5	0.310	*Mycoplasma salivarium*	“Few mouth flora”
7	7	0.248	*Mycoplasma salivarium*	NA
7	12	0.138	*Neisseria sicca*	NA
7	14	0.333	*Ureaplasma parvum*	NA
8	0	0.429	*Staphylococcus aureus subsp. aureus*	NA
9	0	0.351	*Propionibacterium acnes*	No growth
10	0	0.884	*Staphylococcus aureus subsp. aureus*	MRSA
10	1	0.937	*Staphylococcus aureus subsp. aureus*	NA
10	4	0.187	*Staphylococcus aureus subsp. aureus*	NA
11	0	0.512	*Granulicatella adiacens*	“Many mouth flora”
11	1	0.200	*Prevotella melaninogenica*	NA
12	0	0.886	*Pseudomonas aeruginosa*	Moderate *Pseudomonas aeruginosa*
13	0	0.232	*Propionibacterium acnes*	Few yeast
13	1	0.334	*Propionibacterium acnes*	NA
14	0	0.203	*Haemophilus influenzae*	“Few mouth flora”
14	1	0.711	*Haemophilus influenzae*	NA
15	0	0.236	*Propionibacterium acnes*	“Few mouth flora”
15	1	0.960	*Enterococcus faecalis*	NA
15	4	0.960	*Enterococcus faecalis*	NA
15	6	0.898	*Enterococcus faecalis*	NA
15	8	0.928	*Enterococcus faecalis*	Few yeast

For each subject at each timepoint, the proportional abundance of the most abundant lower respiratory tract OTU is depicted, as well as the taxonomic assignment of the OTU as determined by BLAST assignment of a representative sequence from the de novo OTU cluster against the Living Tree Project 16S rRNA gene database. NAs indicate that no clinical culture was obtained during the interval associated with the sample

### 16S rRNA gene sequencing identifies dominant bacteria not found by conventional culture

Two additional subjects who were clinically suspected of having LRTI but had multiple negative clinical cultures obtained from the lower respiratory tract were found to have lower respiratory tract communities dominated by single taxa. Subject 2, with acute myelocytic leukemia (AML) and ARDS, was suspected of developing VAP. 16S rRNA gene sequencing initially revealed mixed taxa consisting mainly of normal LRT constituents (such as *Prevotella*). Concomitant with the clinical suspicion for VAP, LRT sequences became dominated by *Ureaplasma parvum*, which increased in relative abundance to reach 95 % of all LRT sequence reads. In subject 15, with myelodysplastic syndrome and documented RSV infection, LRT 16S rRNA gene sequencing initially revealed mixed taxa, but by day 1 post intubation revealed near-complete dominance by *Enterococcus faecalis*. Neither *Ureaplasma parvum* nor *Enterococcus faecalis* are traditionally recognized as LRTI pathogens in adult critical care patients. Subject 2 was not treated with antibacterials active against *Ureaplasma parvum*, while subject 15 received daptomycin, which is effective against *E. faecalis* but poorly active in the lung.

## Discussion

Analysis of the respiratory tract microbiome in critically ill patients requiring intubation and mechanical ventilation revealed low initial bacterial community diversity compared to healthy controls and a reduction in diversity over time. The clinical diagnosis of LRTI was associated with a trend toward further reduction in alpha diversity of the lower respiratory tract, relative even to those subjects with prolonged courses of intubation. Our results demonstrate features of lower respiratory tract bacterial community structure (i.e., diminished alpha diversity and a single, dominant taxon) that may prove useful in the diagnosis of LRTI in intubated subjects, a group in whom LRTI diagnosis is particularly challenging [36, 37]. The ability of comprehensive microbiome profiling to distinguish between a dominant taxon consistent with LRTI versus a taxon present only at low relative abundance distinguishes this approach from routine bacterial culture, which can confirm the presence or absence of a potential pathogen but provides less information about community structure. Our data also identify unexpected organisms in cases of suspected infection and suggest broadly how 16S rRNA gene sequencing may assist in patient management.

Our study confirms the utility of 16S rRNA gene sequencing as a method to identify pathogens [27–29], including atypical pathogens. When bacterial cultures revealed LRTI pathogens, 16S rRNA gene sequencing confirmed the dominance of the corresponding taxa. In several cases of suspected LRTI with negative clinical cultures, 16S rRNA gene sequencing revealed dominance of unexpected bacteria not typically identified by culture or considered LRTI pathogens. In one of these subjects (subject 2, who was neutropenic), the single dominant taxon was *U. parvum*, an organism not amenable to routine culture but which has been described as a pulmonary pathogen in neonates [38–40]. In the other subject (subject 15, with myelodysplastic syndrome and concurrent RSV-B infection), the single dominant taxon was *E. faecalis*, which is rarely reported as a pulmonary pathogen [41].

Sequence-based analysis of the respiratory microbiome has several other potential advantages in critically ill individuals. (1) Intubated patients typically receive broad spectrum antibiotics, often before cultures are obtained. Antibiotics may turn cultures negative, but since sequence analysis does not depend on bacterial viability, this approach may still identify dominant organisms and allow better targeting of therapy. For example, the failure to detect *E. faecalis* by culture in subject 15 may have resulted from early administration of daptomycin, which could have been sufficient to affect culture results but not to eradicate infection. (2) Respiratory cultures typically exclude normal upper respiratory tract bacteria such as anaerobes, due to the challenges of distinguishing URT contamination from authentic LRTI. In contrast, 16S rRNA gene sequence analysis captures all bacteria present, and relative abundance measures have the potential to reveal outgrowth and dominance by any taxon, including those that would be responsible for anaerobic or URT-derived aspiration pneumonia. Of the subjects sampled at multiple time points, three were suspected of aspiration at the time of enrollment. Two of these subjects showed subsequent emergence of dominant taxa representing normal URT bacteria (*Prevotella* and *Lactobacillus* in subjects 1 and 6, respectively), whereas the third had a dominant organism present in lower abundance and which is less often identified in the URT (*Propionibacterium acnes* in subject 13).

The observation that dominant taxa persist across multiple timepoints, even in the setting of active intravenous antibacterial therapy (e.g., subject 5), raises the question of how this persistence occurs. There are several possible explanations: (1) the resolution of LRTI may require prolonged antibacterial therapy (indeed, present treatment guidelines recommend at least 7 days of treatment) [42]; (2) the recovery of a diverse upper respiratory tract bacterial community may require time even after the successful treatment of LRTI [43, 44]; and (3) sequence-based methods of bacterial community analysis may themselves lag behind ongoing changes by continuing to capture the DNA of dead bacteria. The present study does not allow us to resolve these possibilities, but it is an exciting area for future study.

There are several limitations to this study. (1) Subjects were enrolled after intubation, so the presented data provide no insight into the effect of the intubation itself upon the lower respiratory tract microbiome. (2) While all intubated subjects were sequenced using the Illumina MiSeq platform, the comparison between intubated subjects and healthy controls may be confounded by the use of different sequencing platforms. However, a sub-analysis comparing samples sequenced on both platforms (Additional file 2: Figure S2) suggests that only a small fraction of the difference between groups is attributable to sequencing platform (Procrustes m^2^ = 0.13, *p* < 0.001). (3) Lower respiratory tract samples were obtained by deep endotracheal aspirate in intubated subjects, without concurrent bronchoalveolar lavage available for comparison. Though studies of healthy subjects have consistently shown that the bacterial community composition of the respiratory tract microbiome is similar throughout proximal and distal airway sites [30, 31], the concordance between endotracheal aspirates and direct alveolar sampling in intubated subjects remains a matter of debate, and differences may be exacerbated in the setting of LRTI, where regional heterogeneity of the respiratory tract bacterial community may be increased [45]. (4) Though we attempted to account for antibiotic exposure in our GEE model, it remains an important potential confounder or effect modifier in analysis of the relationship between respiratory tract bacterial community structure and LRTI. (5) The analysis is limited by the lack of a gold standard for LRTI diagnosis and the imperfect sensitivity and specificity of bacterial culture obtained from the lower respiratory tract [46]. (6) Although we suspect that the presence of a single, dominant OTU reflects outgrowth of a single bacterial lineage, proportional abundance may not correlate with absolute abundance; future studies will quantify absolute bacterial abundance by 16S rRNA gene quantitative PCR as well. While molecular analysis offers valuable information not available through traditional respiratory tract culture, it would best serve as a complement to culture, as determination of antibiotic sensitivity currently still requires culture-based analysis, and in some cases species-level identification is not possible from 16S rRNA gene sequence alone.

We considered how patient care might have been affected if the sequence data we obtained had been made rapidly available to clinicians. In subject 5, outgrowth of dominant organism was evident in the sequencing data 5 days before culture results were available—if dominance in the 16S rRNA gene sequence data can be validated as a surrogate for LRTI, these findings would have allowed earlier intervention. In two subjects, unexpected lineages were identified as dominant organisms: *U. parvum* in subject 2, and *E. faecalis* in subject 15. Antibiotic therapy could have been tailored to target these organisms. In addition, detailed early knowledge of dominant organisms would have allowed use of more precisely targeted antibiotic therapies, thereby minimizing activity against the normal microbiota and development of antibiotic resistance.

## Conclusions

In conclusion, we found that longitudinal sampling of the LRT of intubated subjects was feasible, that there were differences among the LRT communities of intubated subjects compared to healthy LRT samples, that features of LRT bacterial community structure correlate with the clinical diagnosis of LRTI, and that 16S rRNA sequencing can identify potential pathogens not detectable by routine culture. At present, optimum detection methods only identify pathogens in 67–81 % of LRTI cases [47, 48], and empiric treatment of LRTI has become the standard of care [49–51]. In an era of rising antimicrobial resistance, the rapid and accurate diagnosis of LRTI and identification of responsible pathogens is essential [52]. Our results suggest that characterization of the respiratory tract bacterial community by 16S rRNA gene sequencing may provide a useful tool in achieving these goals.

## Methods

### Subject enrollment and sample collection

Fifteen subjects were enrolled from the medical intensive care unit (MICU) of the Hospital of the University of Pennsylvania. Initial sample and data collection was performed within 24 h of intubation, with subsequent collection performed at 48- to 72-h intervals thereafter for the duration of mechanical ventilation. Sampling was performed by oropharyngeal (OP) swab and endotracheal (ET) aspirate. Informed consent was obtained from subjects themselves or a patient surrogate. The protocol was reviewed and approved by the University of Pennsylvania IRB (protocol #817706). Healthy controls were non-intubated volunteers without underlying lung disease sampled by OP swab and bronchoscopic bronchoalveolar lavage (BAL) and have been previously described [23, 30].

### Respiratory tract sample collection and management

The OP community was sampled by placing a sterile Copan FLOQSwab against the posterior oropharynx along the external margin of the endotracheal tube. ET samples were collected via the endotracheal tube’s in-line suction catheter. After flushing the suction catheter with approximately 5 mL of sterile saline, the catheter was advanced into the distal trachea and approximately 5 mL of sterile saline was flushed into the trachea and suctioned back into a Lukens trap. All samples were stored immediately on ice and transferred to −80 °C storage within 60 min of collection.

### Nucleic acid extraction, amplification, and sequencing

DNA extraction from OP and ET samples was performed using the MoBio PowerSoil DNA isolation kit [23, 30, 53]. The V1–V2 hypervariable region of the 16S rRNA gene was amplified using barcoded primers 27F (5′-AATGATACGGCGACCACCGAGATCTACACTATGGTAATTGT**AGAGTTTGATCCTGGCTCAG**-3′) and 338R (5′-CAAGCAGAAGACGGCATACGAGATNNNNNNNNNNNNAGTCAGTCAGCC**TGCTGCCTCCCGTAGGAGT**-3′). Underline indicates the Illumina MiSeq adaptor, pad, and linker sequence; bold indicates conserved 16S rRNA gene primer; and Ns indicate the Golay barcode. Thermocycler conditions were as follows: 5 min at 95 °C, 30*(30 s at 95 °C, 30 s at 56 °C, 90 s at 72 °C), 8 min at 72 °C. 16S rRNA gene sequencing was performed via the Illumina MiSeq platform, as described elsewhere [54, 55]. The V1–V2 amplicon was chosen because it permitted robust species-level taxonomic assignment in our prior studies of the lung microbiome [23, 30, 53]. For healthy control subjects, the same region was sequenced via the Roche/454 GS-FLX platform, as described elsewhere [30].

Comparison of control-subject samples sequenced on the Roche/454 platform and intubated-subject samples sequenced on the Illumina MiSeq platform is not optimal. To evaluate the potential confounding by the difference in sequencing platforms (Roche/454 for control subjects, Illumina MiSeq for intubated subjects), a subset of samples from five MICU subjects were sequenced on both the Illumina MiSeq and Roche/454 GS-FLX platforms. The results of taxonomic assignment from 16S rRNA gene sequencing on the two platforms were very similar (Additional file 5: S2A). Procrustes analysis of the weighted, normalized UniFrac distances between samples sequenced on both platforms had an m^2^ of 0.13 (*p* < 0.001), suggesting that only a small fraction of the difference observed between healthy-control samples and intubated-subject samples is attributable to the difference in sequencing platform (Additional file 5: Figure S2B) [56].

Illumina MiSeq sequencing was performed with 250-bp paired-end reads, permitting high-quality coverage of the 316-bp V1–V2 amplicon. Median read lengths were 251 bp from each end; paired reads were filtered to require minimum overlap of 35 bp and maximal difference 15 %. The median number of reads per sample was 37,930 (interquartile range 26,030–92,290) for intubated subjects and 3438 (interquartile range 683–5342) for control subjects. Sterile saline used in sample collection was processed as an extraction and sequencing control, yielding median 62.5 reads per sample by Roche/454 and median 222 reads per sample by Illumina MiSeq.

### Analysis of sequence data

Sequence data was analyzed using the Quantitative Insights Into Microbial Ecology (QIIME) bioinformatics pipeline, version 1.8.0 [57]. Sequence alignment was performed via PyNAST, de novo operational taxonomic unit (OTU) formation and taxonomic assignment via uclust as per QIIME default settings. For comparison to clinical culture data, we evaluated both closed-reference OTUs based on the Greengenes (13.8) taxonomy and de novo OTUs with assignment based on the Living Tree Project database (SSU release 119) [58, 59]. Closed-reference OTUs were used in all comparisons between intubated subjects and healthy controls; de novo OTUs were used in comparison to clinical culture data. Sequence analysis was not performed in real time, and no sequence data was available to clinicians treating the enrolled subjects. (See Additional files 3 and 4: Closed-Reference and De Novo OTU Tables).

### Clinical data collection and analysis

Clinical data including patient diagnosis, physical examination, radiography, laboratory studies, and treatments were extracted from the electronic medical record and patient chart. Antibiotic exposure was coded by functional class, and at each timepoint the presence or absence of gram-positive-, gram-negative-, atypical-, and anaerobic-active agents was assessed. LRTI diagnosis was coded as a binary categorical variable according to the daily attending physician note and subclassified as community/hospital-acquired versus ventilator-acquired depending on whether the attending physician diagnosis was made within the first 48 h of mechanical ventilation. Clinical microbiology information was based on standard respiratory tract cultures collected as per routine MICU protocol; cultures with “mixed flora,” “normal respiratory flora,” or without bacterial growth are interpreted as negative, as per IDSA/ATS guidelines and Center for Disease Control and Prevention surveillance criteria [42]. Data were entered into a REDCap database hosted on secure university servers and exported for analysis into R statistical software, version 3.1.1 [60]. Alpha and beta diversity measures were calculated via QIIME as described in the Results. PERMANOVA testing of pairwise UniFrac and Jaccard distances was performed via the R *vegan* package [61]. Generalized estimating equation modeling was performed via the R *geepack* package’s “geeglm” function [62–64]. The subject ID was incorporated as the cluster variable, the days post intubation as the index of repeated measures, and the correlation structure was specified as “independence” for both models of Shannon index and maximum OTU abundance, based upon visual inspection of each covariance matrix. (See Additional file 5: R Markdown Analysis Outline).

### Availability of data and materials

The data set supporting the results of this article has been submitted to the National Center for Biotechnology Information Sequence Read Archive (SRP062137).

## Additional files

Additional file 1: Figure S1. Comparison of beta diversity among intubated subjects and healthy controls. Principal coordinate analysis was performed on pairwise weighted Jaccard distances for samples from intubated subjects (blue) and healthy control subjects (red), based on sequence read counts aggregated at family-level taxonomy (as in Fig. 2). Panel **A** depicts upper respiratory tract samples. Panel **B** depicts lower respiratory tract samples. The proportion of variance explained by each principal coordinate is noted parenthetically along the horizontal and vertical axes. (PDF 45.8 KB) Additional file 2: Figure S2. Comparison of Illumina MiSeq and Roche/454 sequencing applied to identical samples. (**A**) Subjects 1–5 had upper and lower respiratory tract samples 16S rRNA gene sequencing performed on both the Illumina MiSeq and Roche/454 platforms. A heatmap displays family-level taxonomic assignment (as in Fig. 2). Vertical black lines separate each sample pair, allowing direct comparison of results obtained across the two sequencing platforms. (**B**) Procrustes analysis comparing weighted, normalized UniFrac distances calculated from the closed-reference OTUs formed from each sequencing platform. Balls indicate samples, colored by subject (red—subject 001, blue—subject 002, orange—subject 003, green—subject 004, purple—subject 005); edges connect the results of sequencing on Roche/454 and Illumina MiSeq platforms. Procrustes m^2^ was 0.13 (*p* < 0.001), suggesting similarity of the results across the two sequencing platforms. (ZIP 58.7 KB) Additional file 3:
**Closed-reference OTU table.** Closed-reference OTU taxonomic assignments. Sequence data was analyzed using the Quantitative Insights Into Microbial Ecology (QIIME) bioinformatics pipeline, version 1.8.0. Sequence alignment was performed via PyNAST, closed-reference operational taxonomic unit (OTU) formation, and taxonomic assignment was based on the Greengenes (13.8) taxonomy. Closed-reference OTUs were used in all comparisons between intubated subjects and healthy controls. (ZIP 90.8 KB) Additional file 4:
**De novo OTU table.** Taxonomic assignments of de novo OTUs. Sequence data was analyzed using the Quantitative Insights Into Microbial Ecology (QIIME) bioinformatics pipeline, version 1.8.0. Sequence alignment was performed via PyNAST, with de novo operational taxonomic unit (OTU) formation via uclust as per QIIME default settings. Taxonomic assignment of de novo OTUs was based on BLAST of representative sequences against the Living Tree Project database (SSU release 119), with the highest bit-score chosen. De novo OTUs were used in comparison to clinical culture data. (ZIP 404 KB) Additional file 5:
**R markdown analysis outline.** The code used to read study data, aggregate OTUs for heatmap analysis, determine OTU proportional abundance, evaluate alpha and beta diversity, and perform generalized estimating equations (GEE) modeling of longitudinal data is demonstrated. (RMD 8.30 KB)

## Abbreviations

ALS: amyotrophic lateral sclerosis
AML: acute myelocytic leukemia
BAL: bronchoscopic bronchoalveolar lavage
BMT: bone marrow transplantation
CAP: community-acquired pneumonia
COPD: chronic obstructive pulmonary disease
CX: culture
ESRD: end-stage renal disease
ET: endotracheal
EtOH: alcohol
GEE: generalized estimating equation
GVHD: graft-versus-host disease
HAP: hospital-acquired pneumonia
HCV: hepatitis C virus
HD: hemodialysis
LRT: lower respiratory tract
LRTI: lower respiratory tract infection
MICU: medical intensive care unit
NSCLC: non-small cell lung cancer
OHS: obesity hypoventilation syndrome
OLT: orthotopic lung transplantation
OP: oropharyngeal
OTU: operational taxonomic unit
PE: pulmonary embolism
PEA: pulseless electrical activity
QIIME: Quantitative Insights Into Microbial Ecology
RSV: respiratory syncytial virus
URT: upper respiratory tract
VAP: ventilator-associated pneumonia
