# Supplementary figures and images for: Composition and dynamics of the respiratory tract microbiome in intubated patients
Source: Microbiome. 2016 Feb 11;4:7. doi: 10.1186/s40168-016-0151-8 (PMC4750361; doi:10.1186/s40168-016-0151-8)

### A. Upper Respiratory Tract

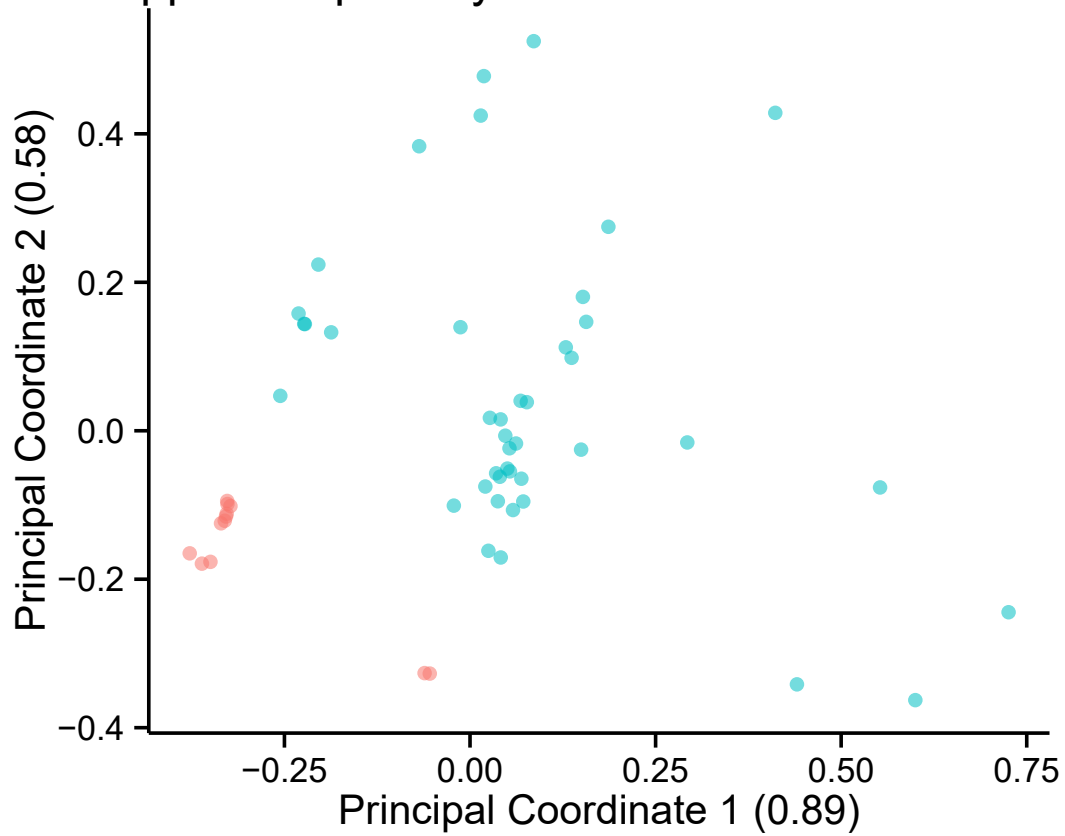

### B. Lower Respiratory Tract

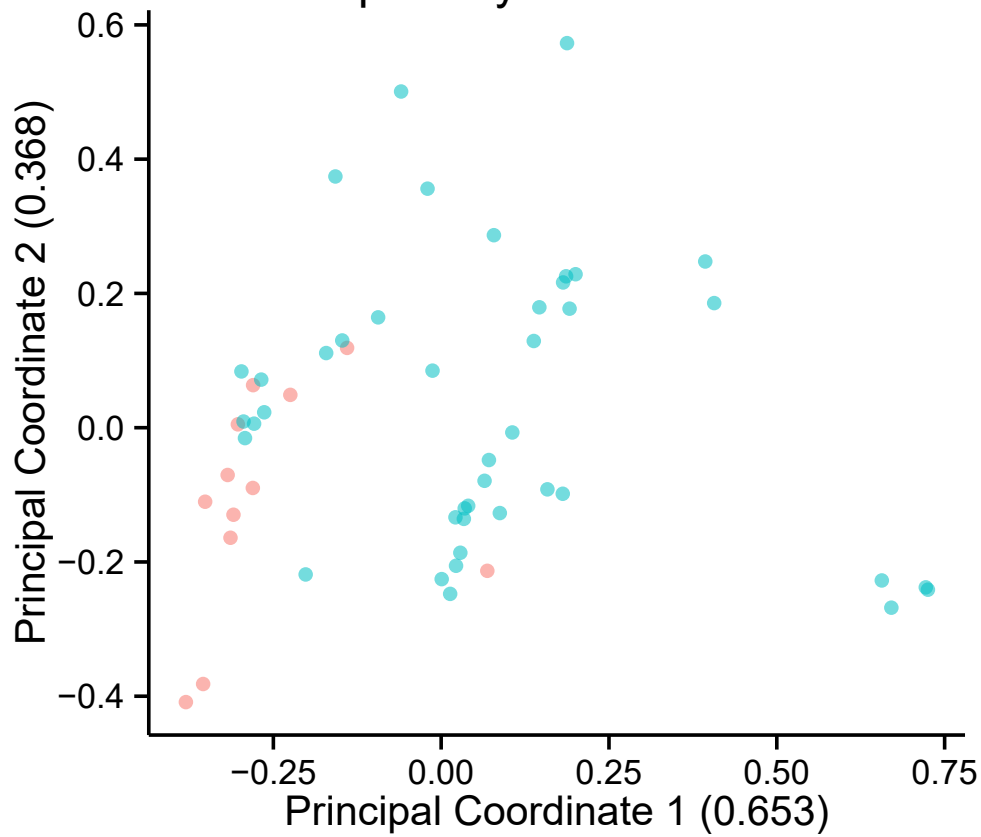

**Subject**    ● Control    ● Intubated

Supplement: Additional file 1: Figure S1. — Comparison of beta diversity among intubated subjects and healthy controls. Principal coordinate analysis was performed on pairwise weighted Jaccard distances for samples from intubated subjects (blue) and healthy control subjects (red), based on sequence read counts aggregated at family-level taxonomy (as in Fig. 2). Panel A depicts upper respiratory tract samples. Panel B depicts lower respiratory tract samples. The proportion of variance explained by each principal coordinate is noted parenthetically along the horizontal and vertical axes. (PDF 45.8 KB) [file 40168_2016_151_MOESM1_ESM.pdf]

# Sequencing Platform Comparison

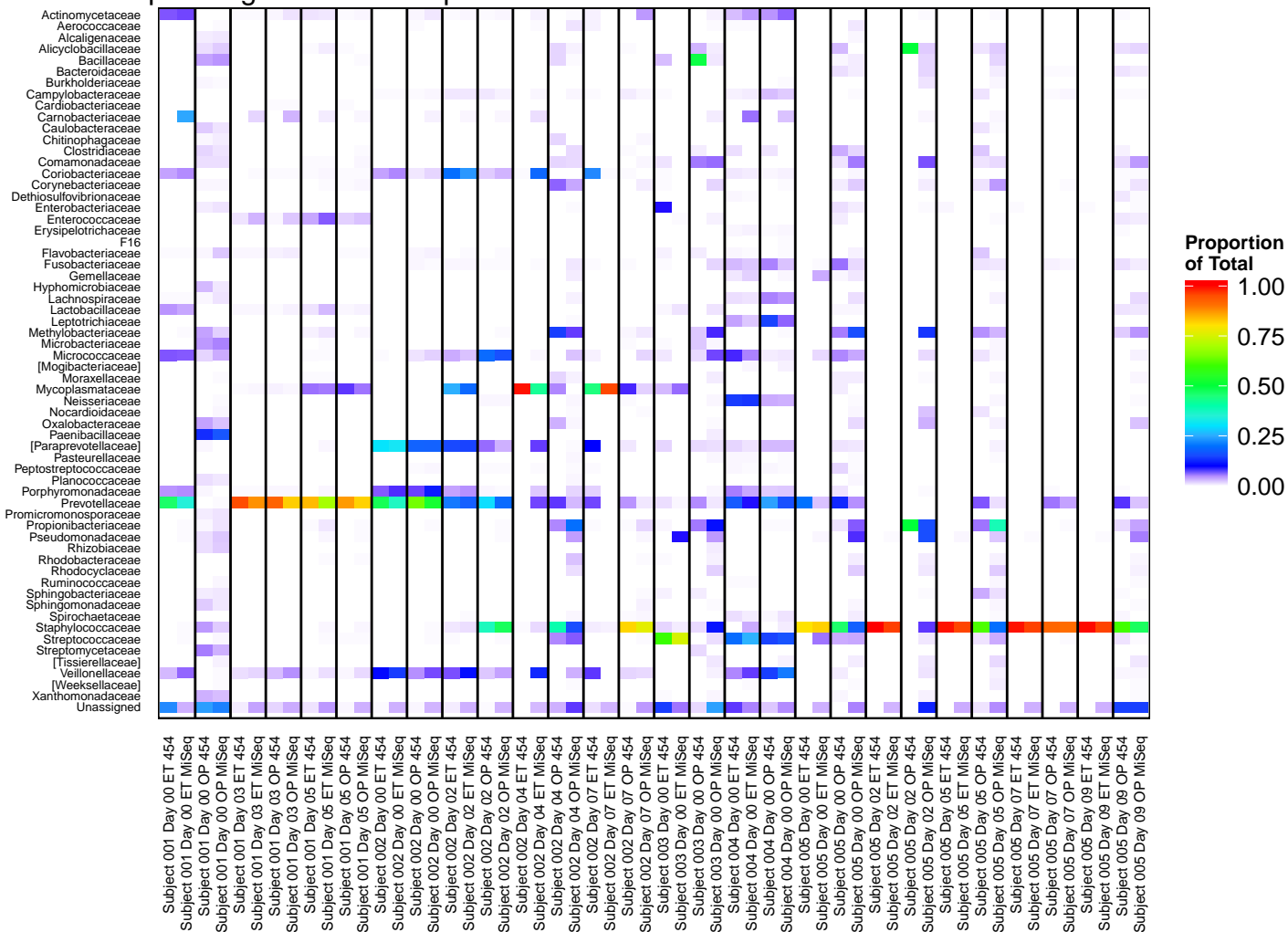

Supplement: Additional file 2: Figure S2. — Comparison of Illumina MiSeq and Roche/454 sequencing applied to identical samples. (A) Subjects 1–5 had upper and lower respiratory tract samples 16S rRNA gene sequencing performed on both the Illumina MiSeq and Roche/454 platforms. A heatmap displays family-level taxonomic assignment (as in Fig. 2). Vertical black lines separate each sample pair, allowing direct comparison of results obtained across the two sequencing platforms. (B) Procrustes analysis comparing weighted, normalized UniFrac distances calculated from the closed-reference OTUs formed from each sequencing platform. Balls indicate samples, colored by subject (red—subject 001, blue—subject 002, orange—subject 003, green—subject 004, purple—subject 005); edges connect the results of sequencing on Roche/454 and Illumina MiSeq platforms. Procrustes m2 was 0.13 (p < 0.001), suggesting similarity of the results across the two sequencing platforms. (ZIP 58.7 KB) [file 40168_2016_151_MOESM2_ESM.zip › figure_supp2A_hm_miseq_454.pdf]

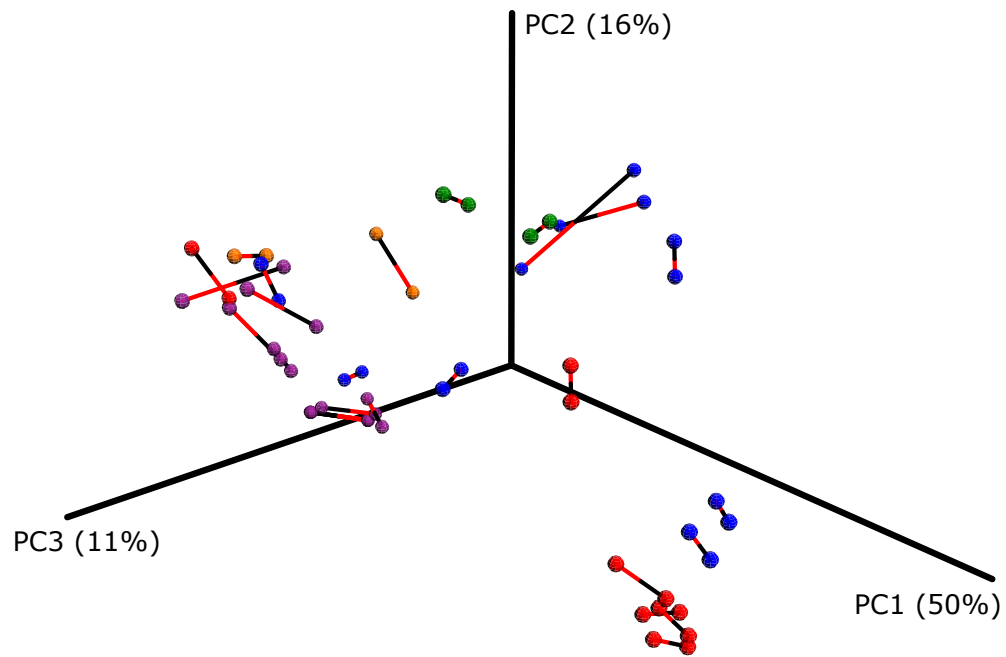

Supplement: Additional file 2: Figure S2. — Comparison of Illumina MiSeq and Roche/454 sequencing applied to identical samples. (A) Subjects 1–5 had upper and lower respiratory tract samples 16S rRNA gene sequencing performed on both the Illumina MiSeq and Roche/454 platforms. A heatmap displays family-level taxonomic assignment (as in Fig. 2). Vertical black lines separate each sample pair, allowing direct comparison of results obtained across the two sequencing platforms. (B) Procrustes analysis comparing weighted, normalized UniFrac distances calculated from the closed-reference OTUs formed from each sequencing platform. Balls indicate samples, colored by subject (red—subject 001, blue—subject 002, orange—subject 003, green—subject 004, purple—subject 005); edges connect the results of sequencing on Roche/454 and Illumina MiSeq platforms. Procrustes m2 was 0.13 (p < 0.001), suggesting similarity of the results across the two sequencing platforms. (ZIP 58.7 KB) [file 40168_2016_151_MOESM2_ESM.zip › figure_supp2B_procrustes.pdf]
